# Continuous renal replacement therapy in critically ill patients does not affect urinary neutrophil gelatinase-associated lipocalin levels

**DOI:** 10.1186/s13054-015-0834-8

**Published:** 2015-03-20

**Authors:** Ioannis Vasileiadis, Chrysoula Pipili, Serafeim Nanas

**Affiliations:** Intensive Care Unit, First Department of Respiratory Medicine, Medical School, University of Athens, Mesogeion 152, ‘Sotiria’ Hospital, 11527, Athens, Greece; First Critical Care Department, ‘Evangelismos’ General Hospital, National and Kapodistrian University of Athens, Ypsilantou 45-47, 10675 Athens, Greece

In the present study, we assessed urinary neutrophil gelatinase-associated lipocalin (uNGAL) during 24-hour continuous renal replacement therapy (CRRT) in critically ill patients. The study was performed in accordance with the Declaration of Helsinki and was approved by the Scientific Council and the Ethics Committee of Evangelismos Hospital in Athens, Greece. Informed consent was provided by family members of the patients included in the study. Eighteen critically ill patients (13 men and five women with a mean age of 69 ± 14 years) in an interdisciplinary intensive care unit underwent continuous veno-venous hemodiafiltration (CVVHD). Three had oliguria (urine output of less than 200 mL per 12 hours). Patient characteristics at enrollment are shown in Table 1.

**Table 1:** **Patient characteristics (n = 18)**

**Patient characteristic**	**Number (percentage) of patients unless noted otherwise**
APACHE II score^a^	19.4 ± 7.5
SOFA score^a^	9.2 ± 2.9
SAPS^a^	69.5 ± 14.2
Reason for admission	
Trauma	1 (5.5%)
Post-operative	11 (61%)
Circulatory	4 (22%)
Respiratory	2 (11%)
SIRS	5 (28%)
Sepsis	4 (22%)
Septic shock	7 (39%)
RIFLE	
No	2 (11%)
Risk	5 (28%)
Injury	10 (55.5%)
Failure	1 (5.5%)
Diabetes	6 (33%)
Vasopressors	11 (61%)

^a^Values are expressed as mean ± standard deviation. APACHE II, Acute Physiology and Chronic Health Evaluation II; RIFLE, risk, injury, failure, loss, end-stage; SAPS, Simplified Acute Physiology Score; SIRS, Systemic Inflammatory Response Syndrome; SOFA, Sequential Organ Failure Assessment.

uNGAL was determined before CRRT onset and at 6 and 24 hours during CVVHD. uNGAL was measured by using a chemiluminescent assay with ARCHITECT technology (Abbott Diagnostics Inc., Abbott Park, IL, USA). For CVVHD, pump-driven machines - from Prisma (a brand of Gambro, Deerfield, IL, USA), Kimal (Dormagen, Germany), or Nephro-Tech (Shawnee, KS, USA) - were used with a 0.9 m^2^ polysulfone filter. uNGAL levels did not change, whereas serum creatinine and serum cystatin C significantly decreased (Table 2). No correlation was found between uNGAL levels and the illness severity scores at inclusion.

**Table 2:** **Renal function biomarkers during continuous renal replacement therapy**

	**Before CRRT**	**At 6 hours**	**At 24 hours**	***P*** **value**
uNGAL, ng/mL	1,917 ± 1,911	1,675 ± 1,634	1,661 ± 1,633	0.23
sCr, mg/dL	2.8 ± 1.5	2.3 ± 0.9	1.9 ± 0.9	<0.0001
sCysC, mg/L	2.74 ± 0.8	1.97 ± 0.64	1.90 ± 0.8	<0.0001

CRRT, continuous renal replacement therapy; sCr, serum creatinine; sCysC, serum cystatin C; uNGAL, urinary neutrophil gelatinase-associated lipocalin.

In a study by Schilder and colleagues [1] in a previous issue of *Critical Care*, plasma NGAL (pNGAL) in critically ill patients was similarly not affected by RRT. No net removal of pNGAL was established, although absorption by the filter with concomitant production could not be definitively excluded. Besides an insufficient clearance through the filter, other causes could be implicated for the remaining NGAL levels.

Whereas pNGAL can come from an extra-renal source (as in sepsis [2]), uNGAL may derive mostly from local synthesis in the kidney [3]. NGAL that has been synthesized in the kidney and can be detected in urine may not efficiently enter the systemic circulation to be cleared by the filter.

Another possible explanation concerns the significance of NGAL function. It has been shown that NGAL has some physiological functions such as carrying out antimicrobial activity and a protective role in the kidney [4,5].

Substituting the function of the kidney with the artificial filter does not mean that the reasons leading to the increased levels of NGAL are eliminated (for example, sepsis or, specifically for the uNGAL, the kidney damage). So regardless of the possibility that the filter cleanses the factor, the production of NGAL might be purposely preserved to carry out its physiological role.

## Abbreviations

CRRT: Continuous renal replacement therapy
CVVHD: Continuous veno-venous hemodiafiltration
NGAL: Neutrophil gelatinase-associated lipocalin
pNGAL: Plasma neutrophil gelatinase-associated lipocalin
RRT: Renal replacement therapy
uNGAL: Urinary neutrophil gelatinase-associated lipocalin

## References

[1] Schilder L, Nurmohamed SA, Ter Wee PM, Paauw NJ, Girbes AR, Beishuizen A (2014). The plasma level and biomarker value of neutrophil gelatinase-associated lipocalin in critically ill patients with acute kidney injury are not affected by continuous venovenous hemofiltration and anticoagulation applied. Crit Care.

[2] Mårtensson J, Bell M, Oldner A, Xu S, Venge P, Martling CR (2010). Neutrophil gelatinase-associated lipocalin in adult septic patients with and without acute kidney injury. Intensive Care Med.

[3] Paragas N, Qiu A, Zhang Q, Samstein B, Deng SX, Schmidt-Ott KM (2011). The Ngal reporter mouse detects the response of the kidney to injury in real time. Nat Med.

[4] Schmidt-Ott KM, Mori K, Li JY, Kalandadze A, Cohen DJ, Devarajan P (2007). Dual action of neutrophil gelatinase-associated lipocalin. J Am Soc Nephrol.

[5] Mishra J, Mori K, Ma Q, Kelly C, Yang J, Mitsnefes M (2004). Amelioration of ischemic acute renal injury by neutrophil gelatinase-associated lipocalin. J Am Soc Nephrol.

